# Patterns of objectively assessed physical activity and sedentary time: Are Nigerian health professional students complying with public health guidelines?

**DOI:** 10.1371/journal.pone.0190124

**Published:** 2017-12-27

**Authors:** Adewale L. Oyeyemi, Suleiman Muhammed, Adetoyeje Y. Oyeyemi, Babatunde O. A. Adegoke

**Affiliations:** 1 Department of Physiotherapy, College of Medical Sciences, University of Maiduguri, Maiduguri, Nigeria; 2 Physiotherapy Department, College of Medicine, University of Ibadan, Ibadan, Nigeria; Universidad Europea de Madrid, SPAIN

## Abstract

**Background:**

Understanding patterns of physical activity and sedentary time is important to effective population-wide primary prevention and control of non-communicable diseases. This study examined the patterns of objectively assessed physical activity and sedentary time, and the prevalence of compliance with physical activity guidelines according to different public health recommendations in a sub-population of health professional students in Nigeria.

**Methods:**

A cross-sectional study was conducted among 102 health professional students (age = 19–34 years old, 43.1% women) of the University of Maiduguri, Nigeria. Participants wore Actigraph accelerometers on their waist for minimum of 5 days/week to objectively measure intensity and duration of physical activity and sedentary time. Prevalence and demographic patterns of physical activity and sedentary time were examined using descriptive and inferential statistics.

**Results:**

The students spent most time in sedentary activity (458.6 ± minutes/day, about 61% of daily time) and the least in vigorous-intensity activity (2.1 ± 4.4 minutes/day, about 0.3% of daily time). Sedentary time was higher among older than younger students (*P*<0.038) and among medical laboratory science students than physiotherapy and nursing students (*P* = 0.046). Total physical activity was higher among nursing and medical students than medical laboratory science students (*P* = 0.041). Although, 85.3% of the students engaged in 150 minutes/week of moderate-to-vigorous physical activity, only 2.9% met the guideline of 75 minutes/week of vigorous intensity activity.

**Conclusions:**

Prevalence of sedentary time was high while that of vigorous-intensity activity was very low among health professional students in Nigeria. Compliance with physical activity guidelines was mainly through accumulation of moderate intensity activity. The results suggest that age and academic programme may influence physical activity level and sedentary behaviour of health professional students in Nigeria. These findings provide preliminary evidence that could be used to inform the needs to develop interventions to improve and support active lifestyle behaviour among students in Nigerian universities.

## Introduction

Physical inactivity is one of the most important modifiable risk factors for many non-communicable diseases (NCDs) including heart diseases, stroke, type-2 diabetes, chronic respiratory diseases and some cancers [1,2]. Almost three quarters of all deaths (28 million) and the majority of premature deaths (82%) from NCDs occur in low- and middle-income countries [1]. In Nigeria, NCDs already account for about 24% of all deaths with prevalence of overweight and obesity at about 33.3% [3]. Thus, controlling NCDs risks through improving the level of physical activity in the population is an important public health priority for Nigeria. However, for effective population-wide intervention, it is important to first identify the patterns of physical activity and sedentariness, and the population sub-groups at risk of physical inactivity [4].

Although the deleterious health consequences and burden of NCDs tend to be expressed later in life, the associated lifestyle and behavioural risk factors including physical inactivity, sedentariness, alcohol consumption and tobacco use tend to be formed during adolescence to early adulthood [5–7]. Since age of university education often coincides with the period of transition to early adulthood, that is critical to establishing independence and adoption of future lifestyles, understanding the patterns of physical activity and sedentariness in university students is relevant to early intervention strategies for improving current and future health in the population. In this context, multiple studies have investigated the prevalence and correlates of physical activity and sedentary behaviours among University students [7–15]. However, most of these studies were conducted in western high income countries and had utilized self-report assessment of physical activity [7–14].

Based on self-report questionnaires, between 22% and 80% of university students globally were physically inactive [11–14], and over 60% did not meet the global recommendations of sufficient health-related physical activity of at least 150 minutes/week of moderate-intensity or 75 minutes/week of vigorous-intensity physical activity, or an equivalent combination [9,10,12,16]. However, when an objective measure of physical activity was utilized, a recent study indicated about 95% of Spanish university students did not meet these global recommendations [15]. Because self-report questionnaires are known to overestimate physical activity [17,18], the exact prevalence of physical inactivity among university students could possibly be higher than the proportion estimated in the previous surveys utilizing self-report measures. Thus, in addition to the utilization of self-report measures, it is important to conduct more studies using objective measures in low-and middle-income countries where the understanding of strategies for promoting physical activity is still poor [19].

To our knowledge, only few studies have been published on physical activity levels of Nigerian university students. These include studies among Nigerian students enrolled in the universities in the United States [20], as part of a multi-country project of health behaviours in 23 countries [13], and conducted primarily to explore correlates of overall physical activity levels among students in Nigerian universities [21,22]. Although these studies suggest moderate to high prevalence of physical inactivity among Nigerian university students, they might have under- or over- estimated the true physical activity status of this population because they were conducted utilizing self-report measures. Thus, for effective health promotion and public health action, it is important to objectively estimate the prevalence and patterns of physical activity among university students and other population sub-groups in Nigeria. Despite the strategic importance of health professionals to promoting physical activity among their patients and in the general population, data regarding physical activity behaviours of health professional students are not readily available. The aim of the present study was to objectively examine the level and demographic patterns of physical activity and sedentary time among health professional students in a Nigeria university. A secondary aim was to examine the prevalence of compliance with physical activity guidelines according to different public health recommendations [16, 23–25].

## Materials and methods

### Study design, setting and participants

A cross-sectional study design was used to collect objective physical activity and self-reported sociodemographic information from health professional students of the College of Medical Sciences, University of Maiduguri, Nigeria. The university is a public and the oldest university in North-Eastern Nigeria. It attracts students from all the 36 states in Nigeria and also international students mainly from the West and Central African countries of Niger and Chad, and Cameroon, respectively. The College of Medical Sciences of the University offers degree programmes in Medicine and Surgery, Dentistry, Nursing, Physiotherapy, Radiography and Medical Laboratory Science (MLS).

The health professional students were selected to participate in the study if they were in the clinical stage of their undergraduate training (from year 4 to 6 for medical and dental students and from year 3 to 5 for the other programmes). They were recruited utilizing convenience sampling technique. A total of 150 health professional students were contacted to participate in the study but only 102 provided valid accelerometer data, giving a response rate of 68%. Eligibility criteria were: (1) living in the students’ residence hall on campus, (2) being between 18 and 39 years, (3) not having any disability that prevented independent walking, and (4) willing to wear accelerometer for 12 hours per day for seven consecutive days. Data were collected from February to May in 2012 and all measurements were done at the University of Maiduguri Teaching Hospital which is the clinical training site for the College of Medical Sciences of the University of Maiduguri.

### Ethics statement

The study was conducted in accordance to the principles expressed in the 1964 declaration of Helsinki and its later amendments. Written informed consent was obtained from all the participants through a cover letter distributed few days prior to the study. The consent procedure and study protocol were approved by the Research and Ethics Committee of the University of Maiduguri Teaching Hospital, Nigeria, (AD/TH/EC/75).

### Measures

#### Assessment of physical activity

Participants wore actigraph accelerometer (Computer Science and Application, Inc. (CSA) Model 7164) activity monitor for seven consecutive days. This device has been shown to provide valid estimate of physical activity [26,27], and correlates highly with heart rate and with other movement and energy-expenditure estimates [28]. Participants were instructed to wear the accelerometer around the waist, just above the right hip, from when they woke up in the morning until bed time at night for the 7 days period. Because the accelerometers were not water resistant, participants were asked to remove them any time they were to perform water related activities like bathing or swimming, and when going to bed. Accelerometer data were considered complete if the participants had valid data of at least 10 hours per day for at least 5 days, including at least 1 weekend day [29,30]. The accelerometers were set to capture the interval of recorded time (epoch) in physical activity at 60 seconds [31,32], and any 60 minutes of consecutive zero counts were considered as non-wear time and screened out of the data [32]. The minute-by-minutes activity counts captured by the accelerometer were collapsed into minutes spent in sedentary, light, moderate and vigorous intensity activities based on the cut-points derived from previous studies [30–32]. A minute of accelerometer data was coded as either sedentary or light if it contained less than 100 activity counts/minute or was between 101–1952 activity counts/minute, respectively. It was coded as moderate if the activity counts were between 1952–5724 counts/minute. Activity counts greater than 5724 counts/minute were coded as minutes in vigorous intensity activity [30–32]. Daily time spent in objective moderate-to-vigorous physical activity (MVPA) was calculated by summing minutes per day of moderate- and vigorous-intensity activity. Total physical activity was calculated as the sum of time spent in light intensity activity and MVPA. The data were scored and interpreted using the “MeterPlus Version 4.2 software from Santech, Inc. (www.meterplussoftware.com).

The participants were classified separately into meeting four guidelines of sufficient physical activity categories and one guideline for sedentary behaviour, based on different public health recommendations [16,23–25]:

- Recommendation 1 (R1): ≥ 150 minutes/week of MVPA [16].
- Recommendation 2 (R2): ≥ 75 minutes/week of vigorous physical activity [16].
- Recommendation 3 (R3): ≥ 30 minutes of MVPA for most days (5 days) of the week [23].
- Recommendation 4 (R4): ≥ 20 minutes/day of vigorous physical activity for at least 3 days/week [24].
- Recommendation 5 (R5): Less than 6 hours/day of sedentary time [25].

Recommendations 1 and 2 are often combined as part of a single guideline [16], but to be consistent with a previous study among university students [15] and to also identify which intensity of physical activity (moderate or vigorous) the students were spending most time to meet the guidelines, we explored R1 and R2 separately in the present study. Further, because there are deleterious metabolic consequences for exposure to 6–10 hours/day of sitting [25], we created a separate fifth recommendation (R5) for sedentary time as less than 6 hours/day.

#### Socio-demographic characteristics

Socio-demographic information of age, gender, marital status, academic programme, level of study and self-rated health status was elicited from the participants. Students' ages were grouped into two categories; 19–24 years, and 25 years and above. Marital status was classified as single/never married and married. Academic programme was classified as physiotherapy, MLS, radiography, medicine and surgery, and nursing. Level of study was categorized into year 3, year 4, year 5 and year 6. Because none of the participants reported poor health status, participants’ self-rated health status was categorized as excellent, very good, and good, while their ethnic group was classified as Hausa/Fulani, Kanuri/Shuwa, Yoruba, Ibo and others.

#### Anthropometrical measurements

Participants’ weight (to nearest 0.5 kg) and height (to nearest 0.1 cm) were measured using a standardized instrument (RGZ-160 Weighing and Height Scale, China). Body mass index (BMI) was calculated as body weight divided by the square of height (kg/m^2^). The participants’ BMI was categorized as underweight (< 18.5 kg/m^2^), normal weight (18.5 –< 25 kg/m^2^), overweight (25 –<30kg/m^2^) and obese (≥ 30 kg/m^2^).

### Statistical analyses

Descriptive statistics of mean, standard deviation and frequencies were calculated for the sociodemographic characteristics of the participants. Physical activity estimates (light-intensity physical activity, MVPA and total physical activity) from the accelerometer were described by mean and standard deviation, in addition to median and interquartile range. Differences in physical activity estimates and sedentary time by demographics (gender, age group, BMI group, level of study and academic programme) were examined by independent t-test and One-way ANOVA statistics as appropriate. The proportion of participants meeting the different physical activity and sedentary time recommendations was computed with percentages, and the gender differences were explored with Chi-Square statistics. All Statistical analyses were performed using SPSS software version 15.0 (SPSS Inc., Chicago. IL).

## Results

The participants comprised of 102 young adults (43.1% female) with a mean age of 23.8 ± 2.3 years and body mass index of 23.3 ± 4.1kg/m^2^. Majority of the participants were single/never married (96.1%), rated their health as ‘very good’ (73.5%), were younger than 25 years (66.7%), and had ‘normal’ weight status (52.9%). Compared to male students, the females were more likely to be overweight (*p <* 0.001) and of Igbo or Yoruba ethnic group (*p* < 0.001) (Table 1).

**Table 1:** Descriptive characteristics of the sample.

Variables	Total Sample (N = 102)	Female (n = 44, 43.1%)	Male (n = 58, 56.9%)	*P*-values†
Age (years)	23.8± 2.3	23.4 ± 1.9	24.2 ± 2.5	0.084
Age group (n, %)				0.258
19–24	68 (66.7)	32 (47.1)	36 (52.9)	
25–34	34 (33.3)	12 (35.3)	22 (64.7)	
BMI (Kg/m^2^)	23.3 ± 4.1	25.1 ± 4.1	21.9 ± 3.7	**<0.001**
Weight status (n, %)				0.053
Underweight	14 (13.7)	5 (35.7)	9 (64.3)	
Normal weight	54 (52.9)	18 (33.3)	36 (66.7)	
Overweight	28 (27.5)	18 (64.3)	10 (35.7)	
Obese	6 (5.9)	3 (50.0)	3 (50.0)	
Marital Status				0.455
Married	4 (3.9)	1 (25.0)	3 (75.0)	
Single/never married	98 (96.1)	43 (43.9)	55 (56.1)	
Ethnicity (n, %)				**<0.001**
Igbo	22 (21.6)	17 (77.3)	5 (22.7)	
Hausa/Fulani	28 (27.5)	9 (32.1)	19 (67.9)	
Yoruba	13 (12.7)	9 (69.2)	4 (30.8)	
Kanuri	12 (11.8)	3 (25.0)	9 (75.0)	
Others	27 (26.5)	6 (22.2)	21 (77.8)	
Acad Programme (n, %)				0.692
Physiotherapy	46 (45.1)	17 (37.0)	29 (63.0)	
MLS	14 (13.7)	7 (50.0)	7 (50.0)	
Radiography	8 (7.8)	3 (37.5)	5 (62.5)	
Medicine & Surgery	20 (19.6)	11 (55.0)	9 (45.0)	
Nursing	14 (13.7)	6 (42.9)	8 (57.1)	
Level of Study (n, %)				0.075
Year 3	16 (15.7)	3 (18.8)	13 (81.3)	
Year 4	49 (48.0)	23 (46.9)	26 (53.1)	
Year 5	35 (34.3)	16 (45.7)	19 (54.3)	
Year 6	2 (2.0)	2 (100.0)	0 (0)	
Health Status (n, %)				**0.022**
Excellent	8 (7.8)	3 (37.5)	5 (62.5)	
Very good	75 (73.5)	38 (50.7)	37 (49.3)	
Good	19 (18.6)	3 (15.8)	16 (84.2)	

†- Values based on independent t-tests statistics for continuous variables and chi-Square Statistics for categorical variables

Acad- Academic; BMI- Body Mass Index; MLS- Medical Laboratory Science

Table 2 shows the daily minutes in sedentary time, light-intensity physical activity, moderate- to- vigorous intensity physical activity and total physical activity. The participants spent 458.6 minutes/day (*SD* = 91.8, Interquartile range [*IQR*] = 393–500, 61.4% of daily valid time) in sedentary activity. Participants spent 288.9 minutes/day (*SD* = 60.7, *IQR* = 257–327, 38.6% of daily valid time) in total physical activity. Of total physical activity, the students spent most of their time in light-intensity physical activity (225.6 ± 48.5 minutes/day, *IQR* = 196–258, about 30.2% of daily valid time), followed by moderate-intensity physical activity (61.3 ± 36.4 minutes/day, *IQR* = 38–81, about 8.2% of daily valid time). The least time was spent in vigorous-intensity physical activity (2.1 ± 4.4 minutes/day, *IQR* = 0.2–1.9, about 0.28% of daily valid time). There was no significant difference (*P* < 0.05) between male and female students on all estimates of physical activity and sedentary time.

**Table 2:** Overall objective physical activity and sedentary time estimates, and comparison by gender.

Variables	Total Sample (N = 102)	Female (n = 44)	Male (n = 58)	*P*-values†
Valid wear days (no.)				0.704
Mean ± SD	6.01 ± 0.83	6.02 ± 0.88	6.09 ± 0.80	
Median (IQR)	6 (5, 7)	6 (5, 7)	6 (5, 7)	
Valid wear time (min/d)				0.805
Mean ± SD	747.5 ± 114.8	735.8 ± 131.9	758.9 ± 101.1	
Median (IQR)	726 (679, 782)	718 (669, 765)	672 (691, 811)	
Non wear time (min/d)				0.564
Mean ± SD	662.7 ± 103.6	669.6 ± 110.2	657.5 ± 99.1	
Median IQR	663 (623, 720)	679 (630, 724)	662 (611, 733)	
Sedentary time (min/d)				0.535
Mean ± SD	458.6 ± 91.8	452.0 ± 96.9	463.5 ± 88.3	
Median (IQR)	452 (393, 500)	443 (394, 482)	459 (389, 516)	
Light PA (min/d)				0.376
Mean ± SD	225.6 ± 48.5	220.7 ± 55.4	229.3 ± 42.6	
Median (IQR)	225 (196, 258)	221 (197, 245)	232 (192, 258)	
Moderate PA (min/d)				0.375
Mean ± SD	61.3 ± 36.4	57.7 ± 28.4	64.1 ± 41.4	
Median (IQR)	61 (38, 79)	63 (31, 81)	60 (43, 78)	
Vigorous PA (min/d)				0.971
Mean ± SD	2.1 ± 4.4	2.1 ± 5.1	2.1 ± 3.8	
Median (IQR)	0.7 (0.2, 1.9)	0.7 (0.1, 2.3)	0.7 (0.2, 1.9)	
MVPA (min/d)				0.390
Mean ± SD	63.4 ± 37.4	59.7 ± 30.2	66.2 ± 42.1	
Median (IQR)	63 (38, 81)	65 (32, 83)	42 (44, 80)	
Total PA (min/day)				0.216
Mean ± SD	288.9 ± 60.7	280.4 ± 65.1	295.5 ± 56.8	
Median (IQR)	288 (257, 325)	285 (257, 319)	291 (256, 339)	

†- Values based on independent t-tests statistics

min/d_ Minutes per day; PA_ Physical activity; *IQR_* Interquartile range; MVPA_ Moderate and vigorous physical activity

There were age group and academic programme differences in time spent in sedentary and total physical activity among the participants. The older age group reported significantly (*P* = 0.038) more sedentary time than the younger age group (480.9 ± 100.4 minutes/day vs. 447.4 ± 85.8 minutes/day). Students in MLS programme spent more time (*P* = 0.046) in sedentary activity (498.3 ± 96.0 minutes/day) than those in physiotherapy (437.2 ± 86.9 minutes/day) and nursing (422.0 ± 58.1 minutes/day) programmes, but less time (*P* = 0.041) in total physical activity (257.2 ± 59.1 minutes/day) compared to those in medicine and surgery (300.7 ± 51.3 minutes/day) and nursing (304.2 ± 44.3 minutes/day) programmes. There was no significant (*P* < 0.05) demographic pattern for light-intensity physical activity, moderate-intensity physical activity and vigorous-intensity physical activity (Table 3).

**Table 3:** Physical activity and sedentary time patterns by age group, BMI, academic program and level of study.

	Sedentary time (min/d)	Light PA (min/d)	Moderate PA (min/d)	Vigorous PA (min/d)	Total PA (min/d)
Age group	Mean ± SD (IQR)	Mean ± SD (IQR)	Mean ± SD (IQR)	Mean ± SD (IQR)	Mean ± SD (IQR)
19–24 years	437.4 ± 85.8 (390, 493)	225.8 ± 52.0 (195, 260)	64.2 ± 41.3 (38, 78)	1.98 ± 4.7 (0.25, 1.96)	291.9 ± 67.2 (260, 336)
25–34 years	480.9 ± 100.4 (405, 535)	225.0 ± 41.2 (195, 248)	55.7 ± 22.9 (37, 80)	2.30 ± 3.8 (0.03, 2.21)	282.9 ± 45.6 (252, 308)
*P*-value	**0.038**	0.936	0.272	0.767	0.485
BMI group					
<25 kg/m^2^	468.1 ± 96.2 (399, 521)	224.8 ± 50.4 (192, 256)	57.5 ± 25.8 (38, 77)	2.20 ± 5.2 (0.06, 1.67)	284.5 ± 55.3 (259, 311)
≥25 kg/m^2^	439.4 ± 80.3 (382, 478)	227.1 ± 45.0 (208, 259)	68.9 ± 51.1 (38, 81)	1.79 ± 2.1 (0.27, 3.14)	297.9 ± 70.3 (249, 345)
*P*-value	0.137	0.817	0.137	0.656	0.295
Level of study					
Year 3	473.5 ± 89.3 (407, 548)	220.0 ± 39.8 (183, 246)	72.7 ± 69.7 (35, 82)	3.10 ± 6.4 (0.25, 1.67)	295.9 ± 75.5 (254, 335)
Year 4	452.5 ± 100.6 (376, 497)	230.4 ± 53.2 (207, 263)	59.9 ± 26.9 (39, 77)	1.91 ± 4.7 (0.14, 1.92)	292.3 ± 63.4 (257, 341)
Year 5 and 6	459.9 ± 82.3 (396, 486)	221.7 ± 45.0 (191, 256)	58.4 ± 25.6 (37, 80)	1.78 ± 2.1 (0.03, 2.10)	281.9 ± 50.6 (254, 317)
*P*-value	0.728	0.627	0.393	0.585	0.652
Acad programme					
Physiotherapy	447.2 ± 86.9 (391, 480)	224.2 ± 55.4 (191, 259)	59.8 ± 47.1 (33, 78)	2.80 ± 5.6 (0.02, 2.92)	286.7 ± 66.9 (255, 314)
MLS	498.3 ± 96.0 (440, 553)	208.7 ± 46.6 (171, 239)	46.4 ± 23.1 (24, 66)	2.10 ± 5.7 (0.03, 0.88)	257.2 ± 59.1 (229, 286)
Radiography	454.3 ± 71.5 (389, 492)	225.9 ± 28.4 (206, 254)	73.6 ± 24.4 (57, 82)	1.70 ± 1.6 (0.20, 3.64)	301.2 ± 37.3 (274, 329)
Medicine & Surgery	477.2 ± 118.4 (372, 567)	234.7 ± 41.8 (210, 260)	64.7 ± 22.3 (43, 80)	1.30 ± 0.9 (0.68, 1.67)	300.7 ± 51.3 (265, 333)
Nursing	432.0 ± 58.1 (386, 466)	233.8 ± 44.1 (206, 252)	69.5 ± 23.9 (54, 94)	0.90 ± 1.0 (0.40, 1.34)	304.2 ± 44.3 (275, 345)
*P*-value	**0.046**	0.591	0.383	0.632	**0.041**

SD = Standard deviation; IQR = Interquartile range. BMI = Body Mass Index; PA *=* Physical activity; MLS = Medical Laboratory Science

**Fig 1:**
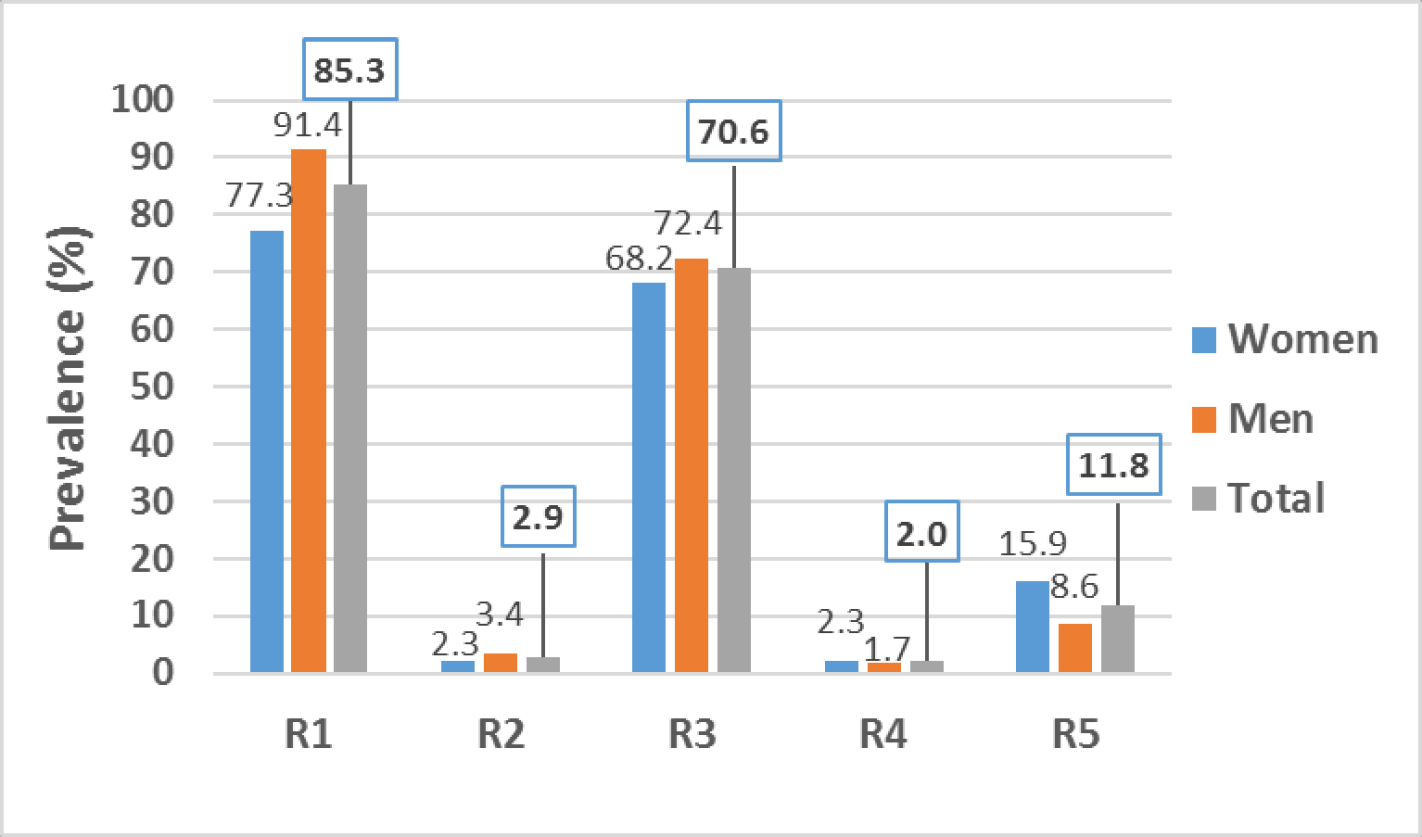
Prevalence (%) of compliance with physical activity guidelines according to different recommendations. R1: At least 150 min/week of MVPA. R2: At least 75 min/week of vigorous physical activity. R3: ≥ 30 min of MVPA for most days (5 days) of the week. R4: At least 20 min/day of vigorous physical activity for at least 3 days/week. R5: Less than 6 hours/day of sedentary time.

The proportion of students meeting the physical activity recommendations based on different public health guidelines is presented in Fig 1. The highest compliant rate was on the World Health Organization (WHO) guideline of 150 minutes/week of MVPA (R1), for which 85.3% (87/102 participants) of the students met the recommendation. More male (91.4%) than female students (77.3%) met the recommendation of 150 minutes/week of MVPA (*P* = 0.047). The Physical Activity Guidelines Advisory Committee (PAGAC) recommendation of at least 30 minutes of MVPA on most days of the week (R3) was met by 72 of 102 participants (70.6%). When the guideline was set at 75 minutes/week of vigorous intensity physical activity (R2), only 3 students (2.9%) of 102 participants met this recommendation. Also, only 2 of 102 participants (2.0%) met the fourth guideline of 20 minutes/day of vigorous intensity physical activity for at least 3 days/week (R4). Finally, only about 12% (12/102 participants) of students met the sedentary time guideline of less than 6 hours of sedentary activity per day (R5). No significant difference exists between the proportion of male and female students meeting R2, R3, R4 and R5 (*P* > 0.05).

## Discussion

Given the future societal roles of health professional students as advocates of healthy lifestyles, it is important to study and understand their physical activity behaviours. This is crucial to establishing regular physical activity habit among future health professionals that would be tasked with the public health responsibility of promoting positive health behaviours in the society. The cohort of Nigerian health professional students in the present study spent more of their time (about 7.5 hours/day) in sedentary activities, much less time in moderate-intensity activities (about 1 hour/day) and a negligible time in vigorous intensity activities (about 2 minutes/day). The findings of high sedentary time and extremely low vigorous-intensity physical activity are consistent with studies of objective physical activity among university and college students in Spain, Portugal and the United States [15,33,34], but different from findings of studies in the general population [35–37]. Also, the time spent in light-intensity activities among students in our study was generally lower than those found in the general population, even among those aged 19 to 34 years (similar age group to our study) [29,35–38]. Health professional students in particular or university students in general, by the nature of their academic work, could be spending longer hours sitting in class to study and may have little time for physical activity compared to other sub-groups in the general population. Taken together, the evidence highlights the needs to institute early health promotion interventions for the establishment and adoption of regular physical activity in university student worldwide.

Although more male than female students met the recommendation of 150 minutes/week of MVPA, there was no significant gender difference in the estimates of physical activity and sedentary time. Such lack of gender difference in objectively determined physical activity and sedentary time estimates was also found among Spanish university students [15]. However, other studies among Portuguese university and American college students have reported male students to be more physically active than their female counterparts, especially on weekdays [33,34]. Although we were unable to explore the variation of physical activity by days of the week (weekdays vs. weekend days), we could speculate that the gender pattern of physical activity among our participants would probably not be different by days of the week. Academic schedules including time-table, class periods and tutorials and non-academic routines like religious obligations at both weekdays and weekend days are ubiquitously similar for male and female health professional students at the College of Medical Sciences in University of Maiduguri. However, since academic schedules are expected to be generally lower during the weekend days than the week days, it is important that future studies among Nigerian university students explore physical activity patterns by days of the week. Possibly, this kind of study could identify subtle socio-cultural issues that may explain the inconsistent findings on gender pattern of physical activity and sedentary time that exist across studies of university students.

Consistent with evidence [4,8,25,39], older age group participants in the present study significantly accumulated more sedentary time than their younger age counterparts. Perhaps, the age of 19–24 years represents the best period to encourage and inculcate physical activity habit among health professional students in the universities. Although, the university period generally coincides with the formative years that is critical to establishing physical activity habit before adulthood, our finding suggests that when designing interventions to improve physical activity behaviours among university students, greater attention should focus on students aged 25 years and above.

We also found programme patterns in the prevalence of sedentary time and total physical activity, with students in the MLS programme accumulating more sedentary time than those in the nursing and physiotherapy programmes but less time in total physical activity than those in medicine and nursing programmes. Perhaps, the MLS students were more sedentary than students in the other programmes because their training requires more sitting activities to prepare slides and cultures for viewing in the laboratory during practical sessions. Compared to students in MLS programme, the nursing, physiotherapy and medical students in Nigeria could spend less time sitting and more time in overall physical activity because their practical and clinic sessions require more standing and walking activities for treatment techniques and procedures, and teaching rounds. A recent study in Nigeria suggests that physical activity levels may be different among Nigerian health professionals with nurses and physiotherapists more likely to be physically active by taking more pedometer steps per day compared to other health professionals [40].

The generally high sedentary time and low vigorous intensity physical activity accumulated by the health professional students in Nigeria should be a call for concern. Evidence indicates that physical inactivity and excessive sedentary behaviour are distinct constructs with independent effects on health outcomes [25, 41]. Since high amount of sitting time has been associated with many chronic diseases risk factors and all-cause mortality [42, 43], spending about 7.5 hours/day in sitting activities by health professional students in our study suggest they are a group at high risk of several chronic conditions. It would be timely to institute practical strategies to reduce the prevalence of high sedentary time among this group of students in Nigeria. Such strategies should include encouraging frequent ‘breaking up’ of prolong sitting time during lectures and other academic activities, and incorporating time out for structured physical activity and exercise as part of their daily or weekly routine. Good evidence exists for such interventions incorporating regular interruption to prolong sitting [25,41]. Also, high level of daily moderate to vigorous physical activity (about 75 minutes/day) have been reported to protect against several cardiovascular risk factors and may eliminate the increased risk of death associated with high sedentary time [44].

Although there is no evidence that the WHO guideline of at least 150 minutes/week of MVPA [16] has a different impact on health than the PAGAC recommendation of at least 30 minutes of MVPA for most days (5 days) of the week [23], both recommendations had the highest compliance rate among Nigerian health professional students. Contrarily, only 2.9% and 2.0% of the students met the recommendations of vigorous intensity activity of at least 75 minutes/week and of at least 20 minutes daily for 3 days in a week, respectively. Taken together, these findings suggest that many Nigerian health professional students did not engage in sufficient vigorous-intensity physical activity but most of them met the MVPA guidelines only through the accumulation of considerable volumes of moderate-intensity activities. Similar accumulations of low volumes of vigorous-intensity physical activity have been reported among university students in Spain and Portugal [15,33]. Since vigorous physical activity could provide greater health benefits than moderate physical activity [44–46], it is desirable to encourage and promote participation in more vigorous-intensity physical activity among university students in Nigeria and elsewhere.

However, our finding regarding majority of the participants meeting the sufficient MVPA recommendations, mainly through accumulation of considerable volumes of moderate intensity activity, should be interpreted with caution. While the public health recommendations for sufficient physical activity were based on at least a 10-minutes bouts of MVPA [16], this 10 minutes bouts of activity was not taken into consideration in our study. Previous studies have shown that the population prevalence of young people complying with sufficient MVPA recommendations could decrease by about 30% to 65% when the 10 minutes bouts criterion is considered [15,35,47]. Thus, the high proportion of health professional students meeting the guidelines of 150 minutes/week of MVPA (85%) and of at least 30 minutes of MVPA on most days of the week (70.6%) could have been substantially lowered if the 10 minutes bouts recommendation was utilized in the present study.

### Limitations and strengths

The relatively small sample size and convenient sampling technique could limit generalization of the findings to other students in different universities in Nigeria. Also, we were unable to stratify the accelerometer data by days of the week making it impossible to explore the variation of physical activity patterns between weekdays and weekends. Future studies with large sample size and accelerometer data stratified by days of the week would allow for more robust analyses to determine weekdays and weekend patterns of physical activity and sedentary time among sociodemographic subgroups of Nigerian university students. However, this study has some important strengths. It was the first to use objective measure of physical activity among university students in Nigeria and one of the very few studies on objective physical activity patterns in the Nigerian population. It provides preliminary evidence on objective physical activity information that could be used to inform the university administrators, health professionals, public health specialists and policy makers on the needs to develop interventions to improve and promote physical activity participation among university students in Nigeria. Moreover, identification of deficits in objectively determined physical activity level of health professional students, in training, may motivate or encourage them to adopt a healthier lifestyle by replacing sedentary behaviour with a more physically active behaviour such as incorporating vigorous intensity activities or aerobic forms of exercise as part of their routines. Since health professionals are generally considered as important health advocates, a carryover of such positive lifestyle behaviour after graduation from the university could inform and contribute to effective promotion of physical activity among their patients and the general public.

## Conclusions

Our findings showed that health professional students in Nigeria spent most of their daily time in sedentary-related activities and a negligible portion of their time in vigorous activities. This cohort of students mostly complied with the physical activity guidelines only through accumulation of moderate intensity activity. The prevalence of sedentary time was higher among older age and MLS students than other students’ groups. The findings suggest the need to institute structured physical activity interventions to minimize sitting time or sedentariness and improve prevalence of vigorous intensity physical activity among health professional students. However, for effective physical activity interventions, future studies should explore the reasons, motives and barriers to participation in physical activity among university students in Nigeria. Overall, the findings provide preliminary evidence that could be used to inform the needs to develop effective physical activity interventions to improve and support active lifestyle behaviour among health professional students in Nigeria. Adoption of healthy behaviour by future health professionals including physiotherapists, nurses, physicians and medical laboratory scientists is crucial to effective primary prevention of chronic non-communicable diseases and health promotion in the underserved and resource constrained health system of a developing country such as Nigeria.

## Supporting information

S1 Appendix Anonymized dataset. (PDF)

## References

[1] World Health Organization. *Global status report on noncommunicable diseases 2014* Geneva: WHO, 2015 10.1161/STROKEAHA.115.008097 25873596

[2] Lee IM, Shiroma EJ, Lobelo F, Puska P, Blair SN, Katzmarzyk PI. Effect of physical inactivity on major non-communicable diseases worldwide: an analysis of burden of diseases and life expectancy. *Lancet*. 2012; 380: 219–229. doi: 10.1016/S0140-6736(12)61031-9 22818936 10.1016/S0140-6736(12)61031-9 PMC3645500

[3] World Health Organization *Noncommunicable diseases (NCD) country profiles*, *2014* WHO 2015 Available at: http://www.who.int/nmh/countries/nga_en.pdf. Assessed December 16, 2016.

[4] Bauman AE, Reis RS, Sallis JF, Wells JC, Loos RJF, Martin BW. Correlates of physical activity: Why are some people physically active and others not? *Lancet*. 2012; 380(9838):258–271. doi: 10.1016/S0140-6736(12)60735-1 22818938 10.1016/S0140-6736(12)60735-1

[5] Spring B, Moller AC, Colangelo LA, Siddique J, Roehrig M, Daviglus ML, et al Healthy lifestyle change and subclinical atherosclerosis in young adults: Coronary Artery Risk Development in Young Adults (CARDIA) study. *Circulation*. 2014;130(1):10–17. doi: 10.1161/CIRCULATIONAHA.113.005445 24982115 10.1161/CIRCULATIONAHA.113.005445 PMC4615574

[6] Ortega FB, Konstabel K, Pasquali E, Ruiz JR, Hurtig-Wennlof A, Maestu J, et al Objectively measured physical activity and sedentary time during childhood, adolescence and young adulthood: a cohort study. *PLoS ONE*. 2013;8(4):e60871 doi: 10.1371/journal.pone.0060871 23637772 10.1371/journal.pone.0060871 PMC3634054

[7] Kwan MY, Cairney J, Faulkner GE, Pullenayegum EE. Physical activity and other health-risk behaviors during the transition into early adulthood: a longitudinal cohort study. *Am J Prev Med*. 2012;42(1):14–20. doi: 10.1016/j.amepre.2011.08.026 22176841 10.1016/j.amepre.2011.08.026

[8] Buckworth J, Nigg C. Physical activity, exercise, and sedentary behavior in College Students. *J Am Coll Health*. 2004;53(1):28–34. doi: 10.3200/JACH.53.1.28-34 15266727 10.3200/JACH.53.1.28-34

[9] Cocca A, Liukkonen J, Mayorga-Vega D, Viciana-Ramírez J. Health-related physical activity levels. *Percept Mot Skills*. 2014;118:247–260. doi: 10.2466/10.06.PMS.118k16w1 24724525 10.2466/10.06.PMS.118k16w1

[10] Varela-Mato V, Cancela JM, Ayan C, Martín V, Molina A. Lifestyle and health among Spanish university students: Differences by gender and academic discipline. *Int J Environ Res Public Health*. 2012;9:2728–2741. doi: 10.3390/ijerph9082728 23066393 10.3390/ijerph9082728 PMC3447583

[11] Haase A, Steptoe A, Sallis JF, Wardle J. Leisure-time physical activity in university students from 23 countries: associations with health beliefs, risk awareness, and national economic development. *Prev Med*. 2004;39(1):182–190. doi: 10.1016/j.ypmed.2004.01.028 15208001 10.1016/j.ypmed.2004.01.028

[12] Irwin JD. Prevalence of university students’ sufficient physical activity: a systematic review. *Percept Mot Skills*. 2004;98:927–943. doi: 10.2466/pms.98.3.927-943 15209309 10.2466/pms.98.3.927-943

[13] Pengpid S, Peltzer K, Kassean HK, Tsala JP, Sychareun V, Müller-Riemenschneider F. Physical inactivity and associated factors among university students in 23 low-, middle- and high-income countries. *Int J Public Health*. 2015;60:539–549. doi: 10.1007/s00038-015-0680-0 25926342 10.1007/s00038-015-0680-0

[14] Keating XD, Guan J, Pin˜ero JC, Bridges DM. A meta-analysis of college students’ physical activity behaviors. *J Am Coll Health*. 2005;54(2):116–125. doi: 10.3200/JACH.54.2.116-126 16255324 10.3200/JACH.54.2.116-126

[15] Arias-Palencia NM, Solera-Martínez M, Gracia-Marco L, Silva P, Martínez-Vizcaíno V, Cañete-García-Prieto J, et al Levels and patterns of objectively assessed physical activity and compliance with different public health guidelines in university students. *PLoS ONE*. 2015;10(11):e0141977 doi: 10.1371/journal.pone.0141977 26536605 10.1371/journal.pone.0141977 PMC4633238

[16] World Health Organization. *Global recommendations on physical activity for health* Geneva, Switzerland: WHO Press; 2010. 26180873

[17] Prince SA, Adamo KB, Hamel ME, Hardt J, Gorber SC, Tremblay MA. Comparison of direct versus self-report measures for assessing physical activity in adults: a systematic review. *Int J Behav Nutr Phys Act*. 2008;5:56 doi: 10.1186/1479-5868-5-56 18990237 10.1186/1479-5868-5-56 PMC2588639

[18] Loney T, Standage M, Thompson D, Sebire SJ, Cumming S. Self-report vs. objectively assessed physical activity: Which is right for public health? *J Phys Act Health*. 2011;8:62–70. 21297186 10.1123/jpah.8.1.62

[19] Hallal PC, Matsudo S, Farias JC Jr. Measurement of physical activity by self-report in low-and middle income countries: More of the same is not enough. *J Phys Act Health*. 2012;9(Suppl1):88S –90S. 10.1123/jpah.9.s1.s88 22287453

[20] Onifade RA. Relationship among attitude, physical activity behavior and physical activity belief of Nigerian students toward physical activity. *Int J Sport Psychol*. 1985;16:183–192.

[21] Adegoke BOA, Oyeyemi AL. Physical inactivity in Nigerian young adults: prevalence and socio-demographic correlates. *J Phys Act Health*. 2008;8(8):1135–1142. 10.1123/jpah.8.8.1135 28796594

[22] Maruf FA, Akosile CO, Umunnah JO. Physical activity, dietary intake and anthropometric indices of a group of Nigerian university undergraduates. *AJPARS*. 2012;4:8–14.

[23] Physical Activity Guidelines Advisory Committee (PAGAC). *Physical Activity Guidelines Advisory Committee Report*, *2008*. Washington, DC, US Department of Health and Human Services, 2008.

[24] Haskell WL, Lee I-M, Pate RP, Powell KE, Blair SN, Franklin BA, et al Physical activity and public health: updated recommendation for adults from the American College of Sports Medicine and the American Heart Association. *Circulation*. 2007;116:1081–1093. doi: 10.1161/CIRCULATIONAHA.107.185649 17671237 10.1161/CIRCULATIONAHA.107.185649

[25] Owen N, Sparling PB, Healy GH, Dunstan DW, Matthews CE. Sedentary behavior: emerging evidence for a new health risk. *Mayo Clin Proc*. 2010;85(12):1138–1141. doi: 10.4065/mcp.2010.0444 21123641 10.4065/mcp.2010.0444 PMC2996155

[26] Brage S, Wedderkopp N, Franks PW. Reexamination of validity and reliability of the CSA monitor in walking and running. *Med Sci Sports Exerc*. 2003;35:1447–1454. doi: 10.1249/01.MSS.0000079078.62035.EC 12900703 10.1249/01.MSS.0000079078.62035.EC

[27] Nichols JF, Morgan CG, Chabot LE, Sallis JF, Calfas KJ. Assessment of physical activity with the Computer Science and Applications, Inc., accelerometer: laboratory versus field validation. *Res Q Exerc Sport*. 2000;71:36–43. doi: 10.1080/02701367.2000.10608878 10763519 10.1080/02701367.2000.10608878

[28] Melanson EL Jr., Freedson PS. Validity of the Computer Science and Applications, Inc. (CSA) activity monitor. *Med Sci Sports Exerc*. 1995;27:934–940. 7658958

[29] Hagstromer M, Troiano RP, Sjostrom M, Berrigan D. Levels and patterns of objectively assessed physical activity—a comparison between Sweden and the United States. *Am J Epidemiol*. 2010;171(10):1055–1064. doi: 10.1093/aje/kwq069 20406758 10.1093/aje/kwq069

[30] Trost SG, Mclver KL, Pate RR. Conducting accelerometer- based activity assessments in field-based research. *Med Sci Sports Exerc*. 2005;37:S531–S543. 16294116 10.1249/01.mss.0000185657.86065.98

[31] Freedson PS, Melanson E, Sirard J. Calibration of the computer science and applications, Inc. accelerometer. *Med Sci Sports Exerc*. 1998;30:777–781. 9588623 10.1097/00005768-199805000-00021

[32] Cain KL, Carrie GM. Accelerometer data collection and scoring manual for adult and senior Studies San Diego State University, San Diego, CA 2012 Available at: http://sallis.ucsd.edu/measures.html. Accessed November 22, 2016

[33] Clemente FM, Nikolaidis PT, Martins FML, Mendes RS. Physical activity patterns in university students: Do they follow the public health guidelines? *PLoS ONE*. 2016;11(3): e0152516 doi: 10.1371/journal.pone.0152516 27022993 10.1371/journal.pone.0152516 PMC4811432

[34] Dinger MK, Behrens TK. Accelerometer-determined physical activity of free-living college students. *Med Sci Sports Exerc*. 2006;38(4):774–779. doi: 10.1249/01.mss.0000210191.72081.43 16679996 10.1249/01.mss.0000210191.72081.43

[35] Baptista F, Santos DA, Silva AM, Mota J, Santos R, Vale S, et al Prevalence of the Portuguese population attaining sufficient physical activity. *Med Sci Sports Exerc*. 2012; 44(3):466–473. doi: 10.1249/MSS.0b013e318230e441 21844823 10.1249/MSS.0b013e318230e441

[36] Dyck DV, Cerin E, De Bourdeaudhuij I, Hinckson E, Reis RS, Davey R, et al International study of objectively measured physical activity and sedentary time with body mass index and obesity: IPEN adult study. *Int J Obes (Lond)*. 2015;39(2):199–207. 24984753 10.1038/ijo.2014.115 PMC4282619

[37] Oyeyemi AL, Oyeyemi AY, Jidda ZA, Babagana F. Prevalence of physical activity among adults in a metropolitan Nigerian city: A cross-sectional study. *J Epidemiol* 2013;23(3):169–177 doi: 10.2188/jea.JE20120116 23604060 10.2188/jea.JE20120116 PMC3700262

[38] Scheers T, Philippaerts R, Lefevre J. Compliance with different physical activity recommendations and its association with socio-demographic characteristics using an objective measure. *BMC Pub Health*. 2013;13:136. 23409982 10.1186/1471-2458-13-136 PMC3599794

[39] Sallis JF, Bull FC, Guthold R, Heath GW, Inoue S, Kelly P, et al Progress in physical activity over the Olympic quadrennium. *Lancet*. 2016; 388(10051):1325–1336. doi: 10.1016/S0140-6736(16)30581-5 27475270 10.1016/S0140-6736(16)30581-5

[40] Owoeye O, Tomori A, Akinbo S. Pedometer-determined physical activity profile of healthcare professionals in a Nigerian tertiary hospital. *Niger Med J*. 2016;57:99–103. doi: 10.4103/0300-1652.182070 27226683 10.4103/0300-1652.182070 PMC4872499

[41] Dunstan DW, Thorp AA, Healy GN. Prolonged sitting: is it a distinct coronary heart disease risk factor? *Curr Opin Cardiol*. 2011;6:412–419. 10.1097/HCO.0b013e3283496605 21785350

[42] Chau JY, Grunseit AC, Chey T, Stamatakis E, Brown WJ, Matthews CE, et al Daily sitting time and all-cause mortality: a meta-analysis. *PLoS ONE*. 2013;8(11):e80000 doi: 10.1371/journal.pone.0080000 24236168 10.1371/journal.pone.0080000 PMC3827429

[43] Biswas A, Oh PI, Faulkner GE, Bajaj RR, Silver MA, Mitchell MS, et al Sedentary time and its association with risk for disease incidence, mortality, and hospitalization in adults: a systematic review and meta-analysis. *Ann Intern Med*. 2015; 162:123–132. doi: 10.7326/M14-1651 25599350 10.7326/M14-1651

[44] Ekelund U, Steene-Johannessen J, Brown WJ, Fagerland MW, Owen N, Powell KE, et al Does physical activity attenuate, or even eliminate, the detrimental association of sitting time with mortality? A harmonised meta-analysis of data from more than 1 million men and women. *Lancet*. 2016,388(10051):1302–1310. doi: 10.1016/S0140-6736(16)30370-1 27475271 10.1016/S0140-6736(16)30370-1

[45] Lakka TA, Laaksonen DE. Physical activity in prevention and treatment of the metabolic syndrome. *Appl Physiol Nutr Metab*. 2007; 32(1):76–88. doi: 10.1139/h06-113 17332786 10.1139/h06-113

[46] Lopez-Martinez S, Sanchez-Lopez M, Solera-Martinez M, Arias-Palencia N, Fuentes-Chacon RM, Martinez-Vizcaino V. Physical activity, fitness, and metabolic syndrome in young adults. *Int J Sport Nutr Exerc Metab*. 2013;23(4):312–321. 23239681 10.1123/ijsnem.23.4.312

[47] Chastin SF, Dall PM, Tigbe WW, Grant MP, Ryan CG, Rafferty D, et al Compliance with physical activity guidelines in a group of UK-based postal workers using an objective monitoring technique. *Eur J Appl Physiol*. 2009;106(6):893–899. doi: 10.1007/s00421-009-1090-x 19488779 10.1007/s00421-009-1090-x

