# Supplementary material for: Patterns of objectively assessed physical activity and sedentary time: Are Nigerian health professional students complying with public health guidelines?
Source: PLoS One. 2017 Dec 27;12(12):e0190124. doi: 10.1371/journal.pone.0190124 (PMC5744983; doi:10.1371/journal.pone.0190124)
Supplement: S1 Appendix — (PDF) [file pone.0190124.s001.pdf]

Anonymized data of the 102 participants

No	Age	Sex	BMI	Level	Program	Valid day	Non wear time	Sed time	LPA	MPA	VPA	MVPA	TPA
1	27	Male	25	Year 5	Physio	7	669	405	260	97	8	105	362
2	23	Male	18.3	Year 3	Physio	6	759	407	247	27	0	27	274
3	20	Male	19.5	Year 3	Radio	6	807	330	218	82	3.8	86	304
4	21	Female	23.1	Year 3	MBBS	7	769	405	179	87	0.7	87	266
5	22	Male	21.1	Year 3	MBBS	6	473	617	285	66	1.7	68	353
6	19	Male	18.2	Year 3	Nursing	6	694	383	258	84	21.7	105	363
7	23	Male	20.4	Year 3	Nursing	7	648	447	272	71	1.3	72	344
8	24	Male	15.7	Year 3	Nursing	6	759	407	247	27	0	27	274
9	22	Female	24.2	Year 4	Physio	7	667	568	198	8	0	8	206
10	22	Female	22.9	Year 4	Physio	7	708	435	269	28	0	28	297
11	24	Female	24.1	Year 4	Physio	7	743	392	229	75	1.7	77	306
12	22	Female	23.9	Year 4	Physio	7	693	444	225	74	3.3	78	302
13	23	Female	18.1	Year 4	Physio	5	814	438	100	17	0	17	116
14	20	Female	18.4	Year 4	Physio	6	656	498	194	90	1.7	92	286
15	22	Female	24.2	Year 4	Physio	6	712	223	93	87	32.5	119	212
16	23	Male	20.5	Year 4	Physio	6	473	614	285	66	1.7	68	353
17	22	Male	23.5	Year 4	Physio	6	689	410	284	58	0	58	342
18	23	Male	19.8	Year 4	Physio	6	662	479	246	51	2	53	299
19	24	Male	17.9	Year 4	Physio	7	682	375	339	43	0.6	44	383
20	24	Male	23	Year 4	Physio	5	861	373	125	78	2.8	80	206
21	22	Male	18.8	Year 4	Physio	7	671	467	264	38	0.1	38	302
22	24	Male	19	Year 4	Physio	5	794	382	181	83	1.3	84	265
23	22	Female	20.2	Year 4	MLS	5	699	435	229	77	0.6	77	306
24	23	Male	16.9	Year 4	MLS	6	570	647	201	21	0.5	22	222
25	21	Male	16.7	Year 4	MLS	7	559	553	275	51	0.6	52	327
26	22	Male	20.4	Year 4	MLS	5	683	507	188	62	0	62	250
27	24	Male	19.6	Year 4	Radio	5	798	365	223	54	0	54	277
28	22	Male	20.3	Year 4	Radio	6	725	470	188	56	0.3	57	245
29	24	Male	21.3	Year 4	Radio	6	613	496	271	59	0.2	60	331
30	21	Male	23.7	Year 4	Radio	7	531	547	232	127	3.4	131	362
31	22	Female	23.5	Year 4	MBBS	7	314	706	308	110	1.1	111	419
32	23	Male	19.2	Year 4	MBBS	5	843	332	188	77	0.3	78	265
33	23	Male	22.1	Year 4	MBBS	6	681	457	233	68	1	69	301
34	23	Male	17.6	Year 4	MBBS	7	648	535	215	42	0.7	42	257
35	24	Male	21.3	Year 4	Nursing	5	751	414	237	38	0.8	39	276
36	23	Female	21.4	Year 5	Physio	5	801	437	180	21	0	21	201
37	24	Female	24.9	Year 5	Physio	6	706	456	269	9	0.2	9	278
38	24	Female	23.5	Year 5	Physio	7	752	425	194	67	2.4	69	263

39	23	Female	24.5	Year 5	Physio	7	637	435	344	24	0.3	24	368
40	23	Male	21.6	Year 5	Physio	5	800	345	221	74	0.5	74	295
41	20	Female	21.1	Year 5	MLS	6	738	397	208	96	0.3	96	304
42	22	Male	22.2	Year 5	MLS	7	632	515	236	55	1.9	57	293
43	24	Female	23.9	Year 6	MBBS	7	595	523	220	99	2.4	102	321
44	22	Male	22.2	Year 5	MBBS	7	651	470	257	60	1.3	61	318
45	23	Male	24.4	Year 5	MBBS	5	666	459	241	71	1.7	73	314
46	23	Female	24.4	Year 5	Nursing	5	787	393	237	23	0	23	260
47	24	Male	31	Year 3	Physio	7	661	566	181	31	0.3	31	213
48	24	Male	31.3	Year 3	Physio	5	698	518	191	322	0.4	322	513
49	23	Female	34.2	Year 3	MLS	7	579	553	226	81	0.4	81	307
50	20	Male	30.7	Year 3	Nursing	5	814	425	162	39	0.3	39	201
51	24	Male	25.4	Year 4	Physio	6	720	420	219	75	4.7	80	299
52	23	Male	25.4	Year 4	Physio	7	731	317	300	87	4.9	92	392
53	22	Male	26.2	Year 4	Physio	7	724	485	218	13	0.1	13	231
54	20	Female	28.5	Year 4	MLS	6	711	348	303	77	0.7	78	380
55	22	Female	26.9	Year 4	MLS	7	651	444	245	96	2.9	99	344
56	24	Female	28.8	Year 4	Radio	5	683	483	205	67	1.4	69	274
57	24	Female	26.9	Year 4	Radio	7	651	463	262	59	3.7	63	325
58	21	Female	29.4	Year 4	MBBS	6	784	308	261	83	4	87	348
59	24	Female	28	Year 4	MBBS	5	562	579	219	78	1.6	80	299
60	22	Female	26.3	Year 4	MBBS	5	731.5	335	302	69	3	72	374
61	22	Female	29.3	Year 4	Nursing	5	815	388	212	24	0.7	25	237
62	23	Male	27.7	Year 5	Physio	5	767	390	214	69	0.3	69	283
63	22	Female	28.9	Year 5	MLS	6	685	472	213	69	1.2	70	283
64	24	Female	25.3	Year 6	MBBS	6	751	443	179	66	0.7	67	245
65	22	Female	29.7	Year 5	MBBS	6	801	368	210	61	0	61	271
66	24	Male	26	Year 5	MBBS	7	624	477	258	78	2	80	338
67	23	Female	33.8	Year 5	Nursing	5	956	352	107	25	0.5	25	132
68	25	Male	19.4	Year 3	Physio	7	669	467	231	56	16.4	72	303
69	27	Male	24.4	Year 3	MBBS	6	504	646	238	49	1.7	51	289
70	25	Female	24.7	Year 3	Nursing	5	694	534	151	60	0.3	60	211
71	25	Male	20.9	Year 4	Physio	6	648	451	258	83	0	83	341
72	27	Male	24.2	Year 4	Physio	6	685	489	217	48	0.2	48	265
73	26	Male	22.2	Year 4	Physio	6	784	351	240	65	0.7	65	305
74	26	Male	16.9	Year 4	MLS	7	426	709	236	68	0.9	69	304
75	27	Female	18.4	Year 4	MBBS	7	684	489	234	33	0.1	33	267
76	26	Male	19.8	Year 4	Nursing	7	648	539	237	15	0	15	252
77	28	Female	17.6	Year 5	Physio	6	683	462	245	49	0	49	294
78	25	Female	20.1	Year 5	Physio	6	434	728	222	44	12	56	278
79	28	Male	20	Year 5	Physio	6	797	376	180	80	5.7	86	266

80	25	Male	19	Year 5		7	648	554	188	45	5.3	50	238
81	23	Female	17.9	Year 4		5	814	438	99	16	0	16	115
82	34	Male	21.1	Year 5		5	775	382	257	26	0	26	283
83	29	Male	23.5	Year 5		6	684	470	192	92	1.2	93	286
84	28	Male	21.1	Year 5		5	649	442	319	30	0	30	349
85	25	Female	23.5	Year 5		7	667	481	208	83	0.7	84	292
86	26	Male	16.3	Year 5		5	830	389	156	36	1	37	194
87	27	Male	23.6	Year 5		5	833	367	175	65	0	65	239
88	26	Male	23.2	Year 5		7	694	486	200	60	0.6	60	260
89	26	Male	26.2	Year 3		5	776	413	217	34	0	34	251
90	25	Male	25.4	Year 3		6	712	459	218	49	0.8	49	267
91	27	Female	25.6	Year 4		7	740	335	312	51	1.6	52	364
92	25	Female	26	Year 4		5	839	343	225	31	0.7	32	257
93	25	Female	27	Year 5		5	726	457	218	38	0	38	257
94	25	Female	27	Year 5		5	726	457	218	38	0	38	257
95	27	Male	25.5	Year 5		7	670	406	259	97	7.7	104	364
96	26	Male	27.3	Year 5		6	685	511	150	94	0.5	94	244
97	25	Female	26	Year 5		5	454	666	288	31	1	32	320
98	28	Female	27.5	Year 5		5	752	453	196	39	0	39	235
99	25	Female	33.8	Year 5		7	662	460	231	81	3.9	85	317
100	25	Male	19	Year 5		7	563	537	187	46	4.3	50	236
101	22	Female	27.1	Year 4		7	652	443	245	97	3.1	100	345
102	25	Male	19.7	Year 5		7	449	617	287	85	0.6	86	373
